# An Agent-based Decision Support for a Vaccination Campaign

**DOI:** 10.1007/s10916-021-01772-1

**Published:** 2021-09-28

**Authors:** Emilio Sulis, Pietro Terna

**Affiliations:** University of Torino - Corso Svizzera 185, 10149 Torino, Italy

**Keywords:** Agent-based modeling, Healthcare support system, Vaccination campaign

## Abstract

We explore the Covid-19 diffusion with an agent-based model of an Italian region with a population on a scale of 1:1000. We also simulate different vaccination strategies. From a decision support system perspective, we investigate the adoption of artificial intelligence techniques to provide suggestions about more effective policies. We adopt the widely used multi-agent programmable modeling environment NetLogo, adding genetic algorithms to evolve the best vaccination criteria. The results suggest a promising methodology for defining vaccine rates by population types over time. The results are encouraging towards a more extensive application of agent-oriented methods in public healthcare policies.

## Introduction

Modelling diffusion phenomena is a subject of increasing interest in many different research areas, e.g. the spread of information in a social context, the supply chain in business process management, as well as the virus diffusion in an environment. The last topic recently received large attention for the practical applications in the context of a pandemic emergency [1]. Modeling efforts can be helpful to address the analysis of the contagions’ sequences exploring alternative scenarios for policy-making.

Three main simulation approaches are System Dynamics (SD) [2], Discrete- Event Simulation (DES) [3], Agent-Based Modeling (ABM) [4]. ABM typically deals with complex systems, where the interaction between multiple actors are neither easily predictable with systems of equations, as in SD approaches, nor with sequences of events, as in DES [5, 6].

Agent-based approaches can also apply Artificial Intelligence (AI) techniques for decision-making, e.g., optimisation with search heuristics, genetic algorithms or reinforcement learning. This paper proposes to apply an AI technique on the top of an ABM concerning the virus spreading by consider- ing where contagions may occur, i.e. the interactions among people and the environment.

In the recent Covid-19 pandemic, the introduction of vaccines cope with the fight against the virus diffusion. In this context, the vaccine distribution policies play a relevant role. The question to address is: which groups should be vaccinated first? Our results suggest how Genetic Algorithms (GA) can be applied to an ABM in order to provide parameter estimates for administering the vaccine to groups of people.

The paper is organised as follows. "Background" reviews the background and the related work. "S.I.s.a.R. model" details the model adopted in this paper. "Research framework and methodology" presents the methodology, while "GA results" introduces GA results. Finally, we conclude the paper in "Conclusions and future work" with some remarks and future work.

## Background

The diffusion processes have been largely studied in different research areas. Typical applications include the spread of innovation [7], the introduction of new products in a market [8], the diffusion of news or rumors in social media by exploring an agent-oriented perspective [9, 10], as well as different real-world social media networks [11]. Healthcare process management benefits from modeling and simulation-based approaches [12–14]. With regard to the virus spread, modeling efforts mostly focused on both systems of equations in complex networks [15] and agent-based approaches [16]. ABM has been widely adopted in public health simulation studies [17], also in vaccine decision-making [18]. In this perspective, ABM investigates complex health behaviour by simulating the actions of individuals influenced by their physical and social environment [19].

*Agent-Based Modeling.* This paper focuses on ABM [20] to study the emergent phenomena [21] in a complex adaptive system [22]. Several ABM toolkits have been proposed in the last decades [23]. One of the most used environment is the free and open-source multi-agent programmable modeling tool NetLogo [6], which have an interesting online application to directly execute models in a Web Browser, i.e. NetLogo Web^1^. Our model reproduces the virus diffusion on a real-world regional scale to explore initial parameter variations (see Section 3). The implementation provides a useful tool to realize *what-if* analysis [24] and represent various scenarios.

*AI techniques for decision-making.* Modelling can be helpful for decision-making to test policy adoption before the effective application. ABM already explored decision support on diffusion processes [25]. In this work, we focus on a system able to set the initial parameters of the model. AI techniques have been largely applied on the top of modeling and simulation [26], also in the healthcare management domain [27]. GA techniques [28] can provide suggestions to the choice of parameters in clinical challenges, by adopting stochastic replicates to sample the responses for a given intervention [29].

## S.I.s.a.R. model

This work focuses on a recent modelling effort to simulate the Covid-19 epidemic diffusion in a region [30]. The NetLogo model (henceforth S.I.s.a.R. model) is publicly available on the Web with an executable version of the simulation program^2^. The model takes its cue from the well-known S.I.R. model [31] that considers three agents’ states: Susceptible (S), Infected (I), and Recovered (R). Similarly, the S.I.s.a.R. model considers four types of agents’ states to better investigate the Covid-19 pandemic, by introducing symptomatic (s) and asymptomatic (a) people, in addition to susceptible and recovered.

Agents are computational entities having several features defined by internal variables. The number of agents for each category are computed from the corresponding frequency distributions in the entire population. For instance, a variable defines the working condition of agents, including categories of interest in the contrast of Covid-19 pandemic, i.e. hospital healthcare operators, nursing home healthcare operators, teachers, students, workers, fragile workers.

The model concerns a reduced scale of 1:1000 of an Italian northern Region (Piedmont), but can be reshaped to simulate other areas. A set of political interventions similar to the real ones, impacts the simulation, e.g., national or local government decisions, restrictions in people movements.

The S.I.s.a.R. model considers people as active agents that can move in the environment, according to their behavioural rules, and if they are allowed for by the policies.

*Agents’ interactions.* A relevant feature of the model involves movements of people (agents), as well as the interactions between an agent and the environment which is at the core of ABM [32].

**Fig. 1:**
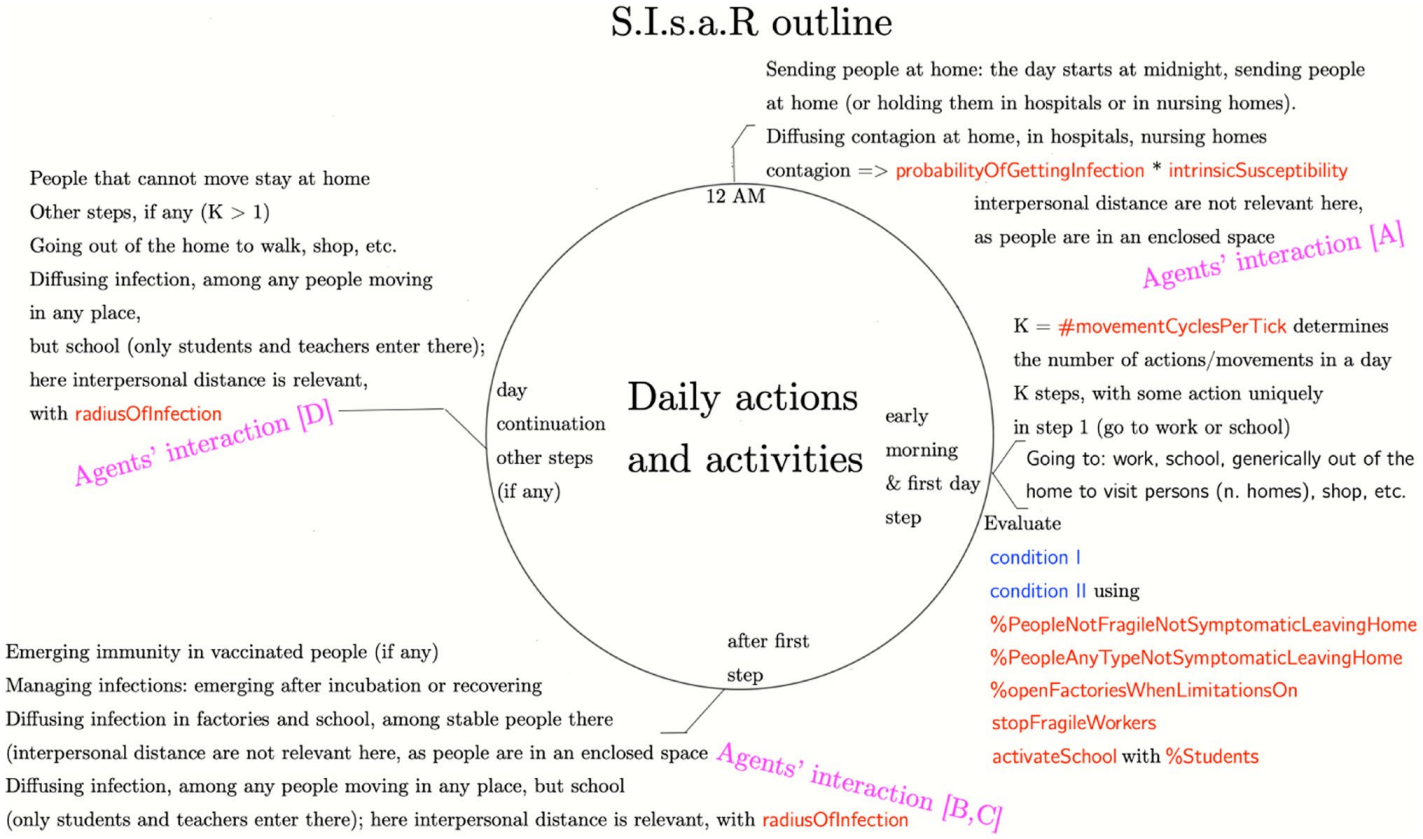
A day in the simulation, with *N* repetition where *N* is the duration of a specific outbreak

Figure 1 describes the daily cycle of the simulation, mentioning the variables used to shape agents’ four types of interactions:

(A) in houses (at night), hospitals, nursing homes;
(B) in schools, workplaces in general, among people stable there;
(C) in the same places (excluding schools) by people temporary there and in open spaces;
(D) interactions mainly in open spaces.

The description of the functioning of the model is out of the scope of the current work, for more details refer to the working document in the project site^3^.

**Fig. 2:**
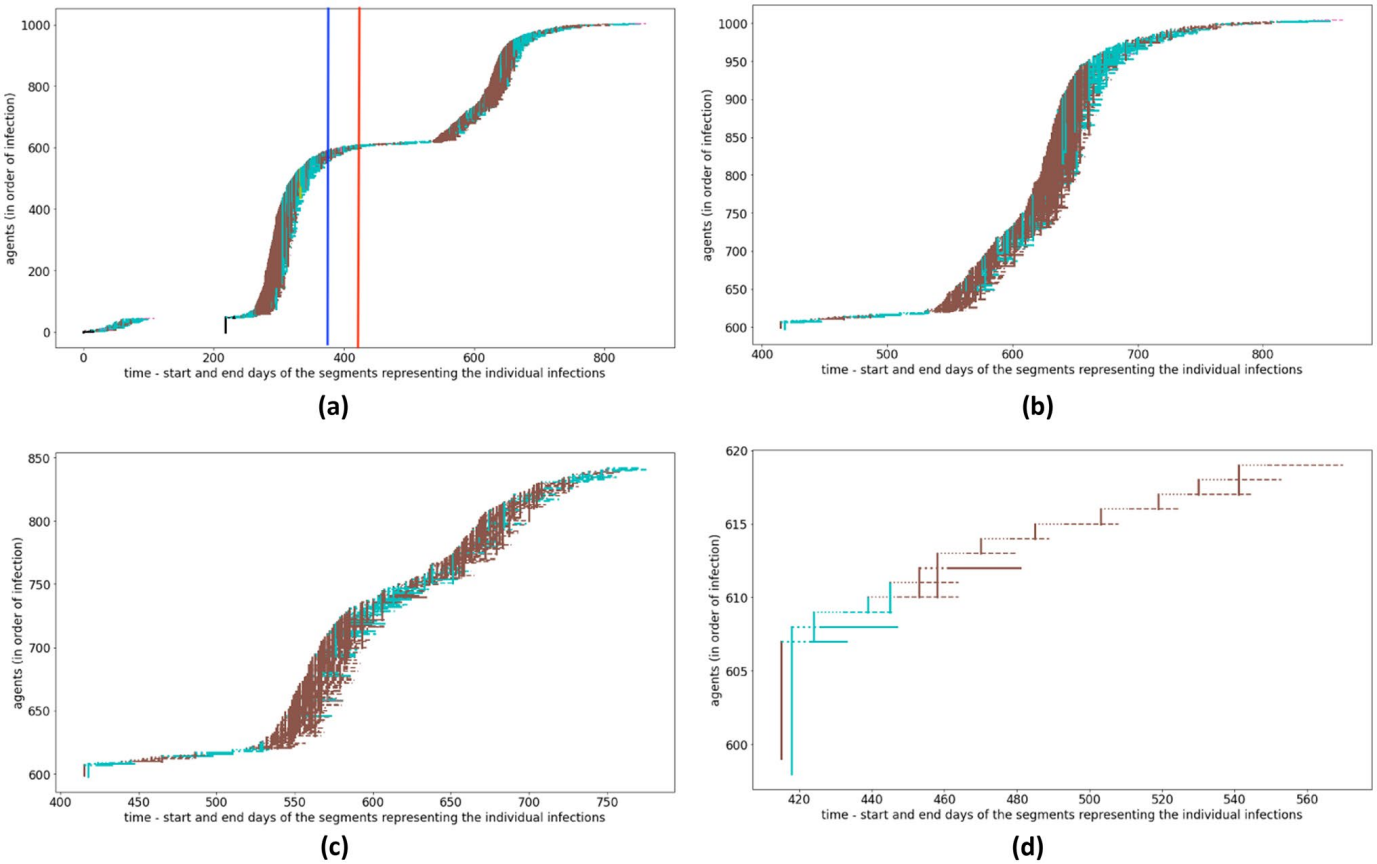
The sequence of contagions in different cases: (**a**) without vaccinations (blue line for the starting point of the vaccination campaign, red line for the start of the effectiveness of the initial vaccinations); (**b**) without vaccinations, after day 413 (**c**) with vaccination campaign (vaccinated people still spreading the infection), after day 413; (**d**) GAs vaccination campaign, with vaccinated people still spreading the infection (best GAs strategy),, after day 413

*Model validation.* To validate the model, we analyse the results of the Covid- 19 simulation in the Piedmont region, started in February 2020. To improve the readability of the outputs, graphic representations describe the infecting agents as an horizontal segment with a vertical connection to another agent receiving the infection, as proposed in Fig. 2.

## Research framework and methodology

The proposed methodology explores the adoption of GA to find optimal parameters of a vaccination campaign on the top of S.I.s.a.R. model. A vaccination campaign makes it possible to immunize large numbers of people. However, the vaccine is not immediately available to the whole population. As a matter of health policy, a choice has to be made about which parts of the population to vaccinate first.

*Modeling vaccine effects.* We know how the vaccine works after a certain amount of time. For instance, between the first and second dose the efficacy is 52%, and the protection concerns starts about twelve days after the first dose [33]. In the model, we compute a delay of 40 days for a vaccine to be effective from the first dose. Once agents have become immune, we simulate a main scenario where they can be contagious (ImmuneInfecting).

*Vaccine administrations.* The goal of the vaccination campaign simulation is to find vaccination sequences by people groups to reduce the number of symptomatic infected people. The S.I.s.a.R. model focuses on a realistic setting, where the vaccination campaign starts after one year since the discovery of the virus. In Italy, the first dose of the vaccine starts the 10^th^ February 2021, i.e. the 373^th^ day since the start of the simulation. New rounds of vaccine administrations occur at regular intervals of about two months. Another relevant date is day 413 (March 22nd, 2021) with the initial effectiveness of the vaccinations. Finally, the execution of the ABM lasts 738 days, i.e., the conclusion is one year after the first dose.

*Selecting optimal parameters.* The population can be divided into categories of interest for the implementation of vaccinations, i.e. seven groups of people (Table 1). In the model, the daily vaccinations quantities are similar to those of Piedmont. The quotas apply to each group to determine the number of vaccination for each day. We start from the first group, which absorbs its quantity; if in that day there are residual vaccine doses, we move to the second group, and so on. The experimental setting concerns the adoption of GA to define the percentage of groups to be involved first. We exploit BehaviourSearch^4^ tool in addition to NetLogo in order to apply GA, with a limit of 300 runs. To increase the computational capabilities we perform GA by using an High Performance Computing infrastructure^5^ [34].

**Table 1:** Categories of persons for vaccine administration

Group	Description
g1	Three sub-categories related to nursing homes: i.health fragile people in nursing homes ii.nursing home operators iii.healthcare operators
g2	Teachers of public and private schools
g3	Workers with medical fragility
g4	Plain workers
g5	Fragile people
g6	Regular people not young not worker not teacher
g7	Young people (excluding fragile cases)

## GA results

The results concerning GA applications have to be compared with the baseline scenario where no vaccine has been introduced. By running the model without any vaccination campaign, at the end of the simulation the number of infected agents is around 325,000, or 7.5% of the whole regional population.

The basis of our work is the agent-based simulation of an epidemic with propagation generated by highly mutable individual agent contacts. This is a model that inherently generates high variability in epidemic trends because even rare sequences of contagions can lead to very different overall outcomes. For this reason, in comparative applications the simulation is performed in repetition batches of ten thousand times and those considered are mean values. The GA uses a subset of cases, carefully chosen as a representative case. Within hundreds of thousands repetitions the extreme cases compensate themselves and also they change quickly.

The set of plots in Fig. 2 details some meaningful results. First, Fig. 2a describes the sequence of contagions without vaccinations (*baseline*). The crucial dates are: the blue line stands for the starting point of the vaccination campaign, while the red line represent the start of the effectiveness of the initial vaccinations. Second, Fig. 2b describes the sequence of contagions after day 413 in the case of no vaccination campaign. The *ImmuneInfecting* scenario is described by the sequence of contagions in Fig. 2c, after day 413, i.e. after the effectiveness of the initial vaccinations. Compared to the *baseline*, the curve is less steep and the total number of infected is lower. Finally, the best GAs strategy is described in Fig. 2d, which is sparse because vaccination works well, and there are few cases and the interval in the abscissa is short.

### A comment on GA results

The simulation in the *baseline* scenario obtains about 325,000 infected cases, while the *ImmuneInfecting* scenario obtains an improvement with a decrease of about 215,000 infected at the end of the simulation. The model with GA selection of groups to be vaccinated first obtains a further improvement reaching about 200,000 infected. The results are similar by excluding symptomatic people when the vaccination campaign effects started (at day 413), as in the second row of Table 2.

**Table 2:** Results of the simulated vaccination campaigns in different scenario. The second row describes the results minus the number of symptomatic people when the vaccination campaign effects started (at day 413)

	At day 413	Baseline	ImmuneInfecting	GAs
TotalFinal TotalFinal—At day 413	197 -	325 128	236 39	**200** **3**

**Fig. 3:**
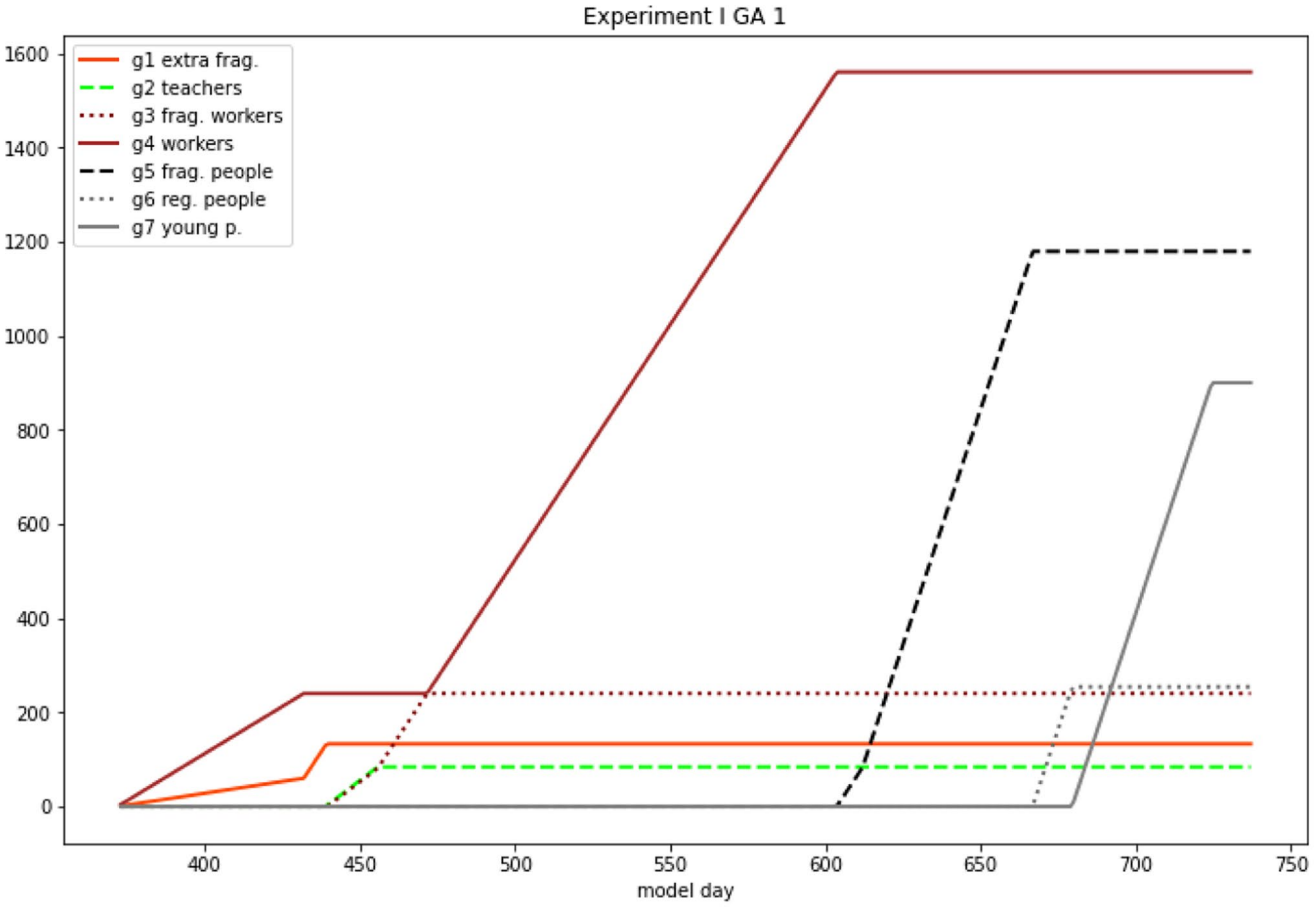
GA vaccination sequence. On the y axis the number of vaccinated subjects of each group. If vaccination is complete, the line is horizontal

The advantage of the GAs strategy is relevant in the realistic case of the vaccinated people still spreading the infection. The main attention of the GAs initially relates to *g4*, and *g6* groups (regular workers and regular persons). They correspond to categories of people who are at risk because they move frequently. Finally, the best GA scenario provides the effects in Fig. 3 on the sequence of groups to be vaccinated first.

## Conclusions and future work

The goal of this work is to suggest how to apply AI techniques on top of ABM to investigate a health decision problem. We described the main steps of a research framework regarding the definition of optimal parameters to address a vaccination campaign. Specifically, we applied GA on a realistic Covid-19 diffusion model. As future work, we plan to improve the scenario analysis by adding cases with different probabilities of infection for immunized persons. We want to explore a *best-checking replicates* test, by adding to GAs the capability to replicate a specific search with the same parameters, but changing the vaccinated people randomly.

## Footnotes

1 See https://netlogoweb.org/

2 See: https://terna.to.it/simul/SIsaR.html

3 See: https://terna.to.it/simul/howSIsaRworks.pdf

4 See: https://ccl.northwestern.edu/netlogo/docs/behaviorspace.html

5 See https://hpc4ai.it/
